# Therapeutic Effect of Yi-Chi-Tsung-Ming-Tang on Amyloid ****β**** **_1−40_**-Induced Alzheimer's Disease-Like Phenotype via an Increase of Acetylcholine and Decrease of Amyloid ****β****

**DOI:** 10.1155/2012/414536

**Published:** 2012-06-13

**Authors:** Chung-Hsin Yeh, Ming-Tsuen Hsieh, Chi-Mei Hsueh, Chi-Rei Wu, Yi-Chun Huang, Jiunn-Wang Liao, Kuan-Chih Chow

**Affiliations:** ^1^Department of Neurology, Show Chwan Memorial Hospital, Changhua, Taiwan; ^2^Graduate Institute of Life Sciences, National Chung Hsing University, Taichung 40001, Taiwan; ^3^Department of Nursing, College of Medicine & Nursing, HungKuang University (HKU), Taichung 40001, Taiwan; ^4^School of Chinese Pharmaceutical Sciences and Chinese Medicine Resources, College of Pharmacy, China Medical University, Taichung 40001, Taiwan; ^5^School of Health, National Taichung University of Science and Technology, Taichung 40401, Taiwan; ^6^Department of Veterinary Medicine, National Chung Hsing University, Taichung 40001, Taiwan; ^7^Graduate Institute of Biomedical Sciences, National Chung Hsing University, Taichung 40001, Taiwan

## Abstract

Alzheimer's disease (AD) is an irreversible neurodegenerative disorder characterized by amyloid accumulation, neuronal death, and cognitive impairments. Yi-Chi-Tsung-Ming-Tang (YCTMT) is a traditional Chinese medicine and has never been used to enhance cognitive function and treat neurodegenerative disorders such as senile dementia. Whether YCTMT has a beneficial role in improving learning and memory in AD patients remains unclear. The present study showed that oral administration of YCTMT ameliorated amyloid-**β**- (A**β**_1−40_) injection-induced learning and memory impairments in rats, examined using passive avoidance and Morris water-maze tests. Immunostaining and Western Blot results showed that continuous A**β**_1−40_ infusion caused amyloid accumulation and decreased acetylcholine level in hippocampus. Oral administration of medium and high dose of YCTMT 7 days after the A**β**_1−40_ infusion decreased amyloid accumulation area and reversed acetylcholine decline in the A**β**_1−40_-injected hippocampus, suggesting that YCTMT might inhibit A**β** plague accumulation and rescue reduced acetylcholine expression. This study has provided evidence on the beneficial role of YCTMT in ameliorating amyloid-induced AD-like symptom, indicating that YCTMT may offer an alternative strategy for treating AD.

## 1. Introduction

Alzheimer's disease (AD) is a progressive neurodegenerative disorder that gradually impairs memory and cognition [1]. It has been estimated that about 5% of the population older than 65 years is affected by AD [2]. The incidence of AD doubles every 5 years and 1275 new cases diagnosed yearly per 100,000 persons older than 65 [3]. In 2010, the number of worldwide AD patients has reached 35 million [4, 5]. The high cost spending on AD treatment and patient care makes AD become one of the most challenging brain disorders in elder human and causes tremendous financial burden.

AD is characterized by cognitive impairment, memory loss, dementia in neuropsychology, and intracellular neurofibrillary tangles and senile plaques in histopathology [6, 7]. During the progress of AD, short-term memory is first affected due to neuronal dysfunction and degeneration in the hippocampus and amygdala. The pathogenic mechanisms of AD include impaired cholinergic function, increased oxidative stress, induction of the amyloid cascade (i.e., amyloid beta, A*β*, deposition, and plaque formation), expression of inflammatory mediators, deficiencies in steroid hormones, and appearance of glutamate-mediated excitotoxicity [2]. The amyloid cascade hypothesis, which suggests a pivotal role for A*β* in the pathogenesis of AD, is accepted by most investigators in this field [8]. A*β* is the major component of the senile plaques [9–11], and extensive studies have indicated that A*β* peptides contribute to the neuronal cell loss and pathogenesis of AD [12, 13].

A*β*_1−40_ is a prone-to-aggregation product of amyloid precursor protein (APP) proteolytic cleavage [14, 15] and has been shown to have a toxic effect on endothelial cells in cerebral circulation in vitro [16]. Direct injection of synthetic A*β*_1−40_  into particular brain regions has been used to induce AD-like pathological changes in animal model [17]. Recently, Passos and colleagues described a mouse model of acute inflammation induced by A*β*_1−40_ intracerebroventricular injection that appears to mimic the early phase of AD progression [18, 19]. Increased oxidative stress owning to lipid peroxidation, protein oxidation, and hydrogen peroxide formation may also be involved in A*β*-induced neurotoxicity [20]. Indeed, antioxidant therapy prevents the learning and memory deficits induced by A*β* in rats [21], and it also delays the clinical progression of the disease in humans [22]. Proteomic analysis using peptide mass fingerprint (PMF) has also revealed A*β*_1−40_-induced changes in protein expression in the rat hippocampus [23].

Acetylcholine (Ach), a neurotransmitter of cholinergic system, influences neuron plasticity and plays an important role in cognition and memory [24, 25]. A*β* deposition is closely associated with dysfunction and degeneration of cholinergic neuronal circuits in the basal forebrain nuclei and also results in deficit of Ach in cortical and hippocampal areas [26]. Consistently, upregulation of acetylcholinesterase, an enzyme that catalyzes the hydrolysis of Ach, has been reported within and around amyloid plaques [27], and upregulation of its activity has also been shown to encourage the assembly of A*β* into fibrils that lead to A*β* toxicity [28]. The level of Ach has also been shown to be related to the degree of amnesia and A*β* depositions in the AD brain [29]. All these studies suggest that Ach plays an important role in AD [30, 31].

While up to now there has been no effective treatment for AD, targeting A*β* production and reversing Ach diminution are attractive therapeutic strategies for AD [32, 33]. In addition, alternative medicines that improve AD symptom have also been recently identified and they are natural and may be safer and more effective than currently commercialized drugs for AD [32, 33]. YCTMT was first introduced by an ancient Chinese physician, Dong-Yuan Li, in Jin Dynasty (AD 1200s). YCTMT is a decoction made of various Chinese herbs including *Astragali radix*, *Ginseng radix*, *Puerariae radix*, *Paeoniae lactiforae*, *Phellodendri contex*, *Viticis fructus*, *Cimicifugae uralensis*, and *Glycyrrhiza uralensis*. Ginseng can reduce oxidative stress that damages neuroplasticity, neurogenesis, and memory formation [34, 35]. In addition, Ginseng has also been shown to promote axonal and dendritic extension [36]. Astragalus promotes axonal maturation and prevents memory loss in mice [37]. Puerarin and Glycyrrhiza have been shown to have neuroprotective effects against A*β* treatment in mouse model [38, 39]. According to the theory of Dr. Li, Yi-Chi-Tsung-Ming-Tang (YCTMT, also known as Yiqicongming decoction) treats or prevents dizziness, tinnitus, and blurred vision. Therefore, it is intriguing to know whether YCTMT also has a beneficial effect on ameliorating AD-related symptoms. The aim of this study was to evaluate the effect of YCTMT treatment on A*β*_1−40_-induced AD-like symptoms and possible underlying mechanism.

## 2. Materials and Methods

### 2.1. Drugs and Reagents

YCTMT is composed of *Astragali radix*, *Ginseng radix*, *Puerariae radix*, *Paeoniae lactiforae*, *Phellodendri contex*, *Viticis fructus*, *Cimicifugae uralensis*, and *Glycyrrhiza uralensis* in a ratio of 5 : 5 : 5 : 1 : 1 : 1.5 : 3 : 5 (dry weight). All components were purchased from a Chinese herbal shop in Taichung city, Taiwan, and confirmed by Professor Ming-Tsuen Hsieh. YCTMT (0.5 kg) was prepared as a mixture of all above components and extracted with 5 L distilled water at 100°C. The liquid extract was powdered by heating in a rotary vacuum evaporator. In the study, YCTMT powder was dissolved in distilled water to make the final concentrations at 0.5, 1.0, and 2.0 g/mL. A*β*_1−40_ was purchased from Tocris Bioscience (Ellisville, MO, USA) and dissolved in a vehicle containing 35% acetonitrile and 0.1% trifluoroacetic acid. Other chemicals were purchased from Sigma-Aldrich (St Louis, MO, USA).

### 2.2. Animals

Adult male Sprague-Dawley (S.D.) rats (200–250 g) were purchased from BioLASCO (Taipei, Taiwan). The rats were maintained on a standard diet with water *ad libitum* and housed under a 12 : 12 light-dark cycle in a temperature-controlled environment (23 ± 1°C). The animals were cared in accordance with guidelines provided by the Institutional Animal Ethics Committee of China Medical University (Taichung, Taiwan).

### 2.3. Rat Model with AD-Like Phenotype

**Figure 1:**
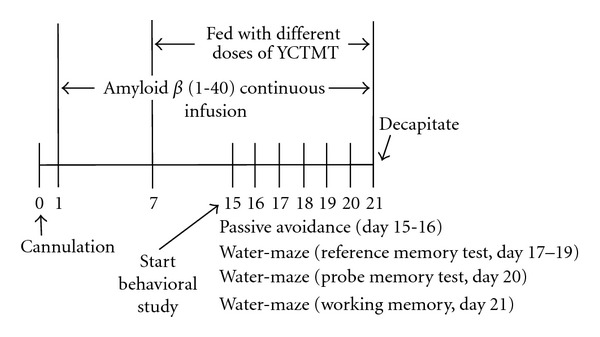
Experimental scheme.

Rat model with AD-like phenotype was developed by infusing A*β*_1−40_ into cerebral ventricle in the brain as described previously [18, 19, 40]. Briefly, one week before experiment, animals were randomly divided into five groups (*n* = 9 for each group). At day 0, rats were anaesthetized with phenobarbital (45 mg/kg, i.p.), placed in a Narishige stereotaxic instrument with the head being fixed. The skull was opened carefully, and a cannula was implanted into the right ventricle at coordinates: A − 1.4,   L ± 2.4, and V 7.2, using an atlas [41]. At day 1, vehicle or A*β*_1−40_ was continuously administered (25 pM/day) intraventricularly at an infusion rate of 0.5 *μ*L/h for three weeks via the cannula that was powered by a miniosmotic pump (model 2002, Alzet, CA, USA). The control rats were infused with the vehicle only. From the 8th day, rats were fed with YCTMT (0.5, 1.0, and 2.0 g/kg/day) orally once a day. The experimental scheme was presented in Figure 1.

### 2.4. Passive Avoidance Test

The passive avoidance test was performed at day 15 and 16 after surgery according to step-through passive avoidance task previously described [42]. Briefly, the guillotine door connecting the light and dark space was closed during the trial training. When a rat was placed in the light space, with its back facing the guillotine door, the door was opened. The time of step-through latency (STL) taken by the rat to enter the light space was measured with a stopwatch. Once the rat entered the dark space, the door was closed. An inescapable scrambled foot-shock (1.0 mA for 2 s) was then delivered through the grid floor. The rat was removed from the dark space 5 seconds after the shock was administered. Then, the rat was put back into the home cage until the retention trial was over.

### 2.5. Morris Water-Maze Test

A Morris water-maze test was used in the study to assess the memory capability [43, 44]. In brief, the apparatus consisted of a circular water tank (180 cm in diameter, 60 cm in height, filled with water in a depth of 50 cm, at 28 ± 1°C) with a platform (11 cm in diameter) set under the water. Morris water-maze is a swimming-based model in which animals must learn to escape from water and step onto the platform. An escape platform was placed in a constant position in the center of one of the four quadrants of the tank and 1 cm below the water surface. For reference memory test, the rats were trained for 3 days (starting from day 17 to 19), 120 s per trial, and 4 trials per day starting at four different positions with 30 min intervals (Figure 1). If the rats could not find the hidden platform within each training session (120 s), the animals were led to it. If the rats swam onto the platform within 120 s, the rat was allowed to stay on the platform for 30 s then returned to home cage. In each training session, the latency to escape onto the hidden platform was recorded. For probe memory test, the hidden platform was removed on day 20, and memory retrieval was examined by a probe trail that lasted for 120 s in the pool. The time at which the animals crossed the annulus where the platform had been located was assigned. Twenty-four hours after probe trial, working memory (reacquisition) test was performed by measuring the time each rat spent in the new quadrant of the target platform.

### 2.6. Immunohistochemical Detection for A*β* and Ach Expression in Brain

Rats were sacrificed at day 21 with pentobarbital (90 mg/kg, i.p.) and perfused transcardially with saline, followed by 4% paraformaldehyde in saline. After postfixation, the whole brain was removed and prepared for paraffin slice. Brain sections with 5 *μ*m thickness were obtained on a microtome (Leica 2030 Biocut) at 1-mm interval from the stereotaxic coordinates between −1.46 mm and −3.40 mm Bregma. Brain sections were deparaffinized in xylene, rehydrated in a series of ethanol, and endogenous peroxidase quenched with 1% (v/v) H_2_O_2_ in methanol and microwaved for 15 min (with 650 W) in 0.01 M citrate buffer (pH 6.0). Nonspecific binding sites were blocked by blocking buffer containing 10% (v/v) goat normal serum and 0.1% Triton X-100 in PBS for 60 min at room temperature. Antibodies against A*β* (1 : 300 dilution, Convance, no. SIG-39220) and Ach (1 : 300 dilution, Chemicon, Billerica, MA, USA) were applied to sections overnight at 4°C. Sections were washed with PBS, incubated with biotinylated secondary antibody for 2 hr at 25°C, washed, and placed in avidin-peroxidase conjugate solution for 1 hr. The horseradish peroxidase reaction was detected with 0.05% diaminobenzidine and 0.03% H_2_O_2_. The reaction was stopped with H_2_O, and sections were dehydrated in an ethanol series, cleared in xylene, and coverslipped in permanent mounting solution. Protein expression area in each brain section was measured according to Lim et al. [45] and Liao et al. [46] with slight modification. Briefly, a full brain middle section area was measured by an image analyzer (Leica, Q500MC, Nussloch, Germany). Brain sections of each rat group were measured under 40x magnification, and at least 20 fields from each brain section were counted. The permillage of A*β*- or Ach-positive areas was calculated using the following equation: ‰ of A*β* or Ach area = (sum of A*β*- or Ach-positive area/total area of brain section) ×1000‰.

### 2.7. Western Blot

Protein extraction was performed in RIPA lysis Buffer 50 mM Tris, pH 7.4, 150 mM NaCl, 1% NP-40, 0.5% sodium deoxycholate, 0.1% SDS, protease inhibitor cocktail (sodium orthovanadate, sodium fluoride, EDTA, leupeptin), and 1 mM PMSF. Quantification of protein was performed using the BCA Protein Assay Kit (Beyotime). Equal amounts of protein samples were separated on 12% SDS-PAGE gels and transferred onto a PVDF membrane at 0.8 mA/cm^2^ for 1.5 h. The membrane was blocked with 5% nonfat milk in a 20 mM Tris-HCl (pH 7.4) containing 150 mM NaCl and 0.05% Tween20 (TBS-T) for 1 h at RT and incubated with the primary antibodies: *β*-amyloid antibody (1 : 1000 dilution, Abcam, Cambridge, MA, USA), antiacetylcholine (Chemicon, no. MAB5302.), and *β*-actin (abs, no. abs-24) at 4°C overnight. After being washed with TBS-T, the membrane was incubated with HRP-conjugated IgG (MILLIPORE, Chemiluminescent HRP Substrate, no. WBKLS0500) for 1 h at RT, washed with TBS-T again, and detected by ECL (Thermo Scientific). Densitometric quantitation was analyzed with FUJIFILM, Multi Gauge V3.0 Software, and standardized with *β*-actin. All Western Blotting samples were run in triplicate.

### 2.8. Statistical Analysis

All of the data obtained were expressed as mean ± standard errors of means (SEM) and analyzed using one-way analysis of variance (ANOVA), followed by post hoc between-group analyses using Scheffé's test for multigroup comparisons. The criterion for statistical significance was *P* < 0.05 in all evaluation.

## 3. Results

### 3.1. YCTMT Posttreatment Reversed A*β*_1−40_-Mediated Impairment in Memory Retention

**Figure 2:**
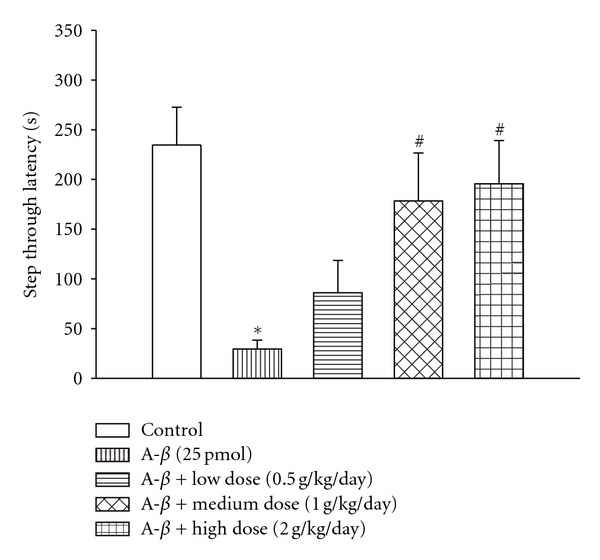
Passive avoidance test at day 15 after A*β*_1−40_ infusion. A*β*_1−40_ infusion reduced step-through latency of rats, and it was restored by YCTMT posttreatment (*n* = 9 for each group). YCTMT (0.5, 1.0, and 2.0 g/kg/day) was administrated for a week before test. *Represents significant difference between the indicated and normal control group; ^#^between the indicated and A*β*_1−40_ group, *P* < 0.05.

Result from passive avoidance test showed that at day 15 after A*β*_1−40_ infusion significantly reduced the step through latency (STL) of treated mice compared to the vehicle injection (*P* < 0.05, Figure 2), suggesting an A*β*_1−40_-induced impairment in memory retention after 2-week continuous injection. YCTMT posttreatment for 7 days with doses of 1.0 and 2.0 g/kg/day, but not 0.5 g/kg/day, significantly attenuated A*β*_1−40_-mediated reduction in STL (*P* < 0.05, Figure 2), indicating that YCTMT reversed A*β*_1−40_-mediated impairment in a dose-dependent manner. There was no significant difference between the control and YCTMT-treated groups at 1.0 and 2.0 g/kg/day. This result suggested that treatment with YCTMT recovered A*β*_1−40_-induced deficit in memory retention in rats.

### 3.2. YCTMT Effectively Reversed A*β*_1−40_-Induced Impairments in Learning and Memory

**Figure 3:**
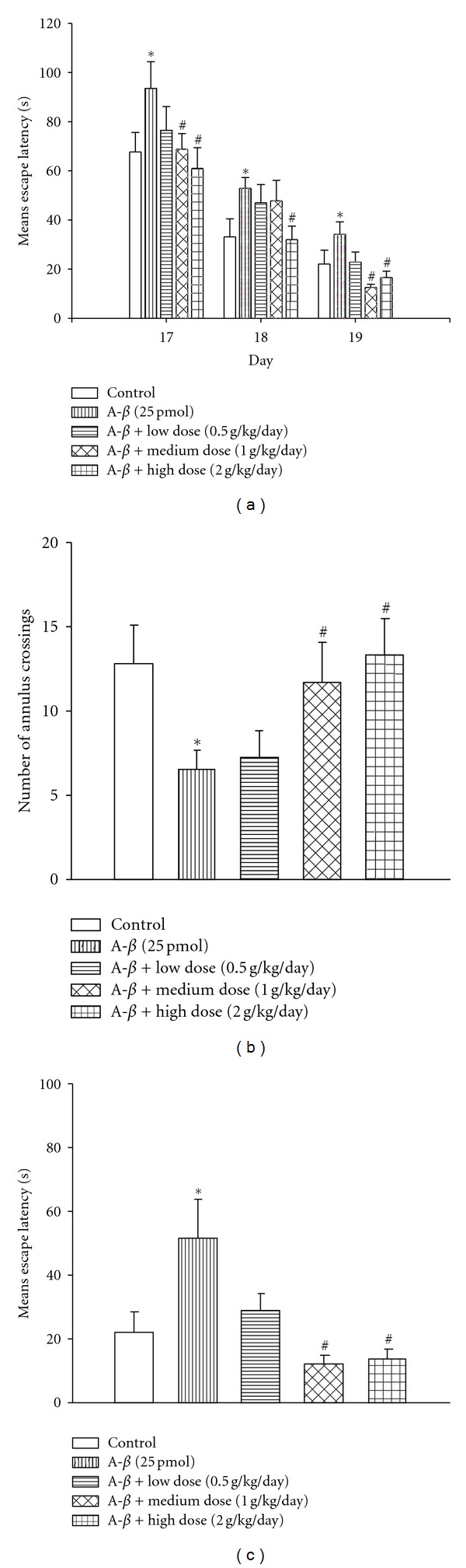
YCTMT posttreatment improved memory of A*β*_1−40_-infused rat in Morris water-maze test. (a) Reference memory test at day 17–19. (b) Probe test at day 20. (c) Working memory test at day 21. A*β*_1−40_-infused rats were administrated with YCTMT (0.5, 1.0, and 2.0 g/kg/day) before tests. *Represents significant difference between the sham and A*β*_1−40_ on the same day; ^#^is for the comparison between A*β*_1−40_ and A*β*_1−40_ + YCTMT on the same day, *P* < 0.05.

To further evaluate the therapeutic effect of YCTMT on A*β*_1−40_-induced impairments in learning and memory, Morris water maze test was conducted. In reference memory test, these A*β*_1−40_-treated animals showed significantly longer escape latency than normal control animals at day 17, 18, and 19 postinfusion of amyloid (Figure 3(a)). Posttreatment of YCTMT with the lowest dose of 0.5 g/kg/day showed no effect on A*β*_1−40_-caused deficits. However, YCTMT treatment at dose of 1.0 g/kg/day significantly reduced escape latency in A*β*_1−40_-treated rats at day 17 and 19, but not at day 18. Posttreatment with 2.0 g/kg/day YCTMT reversed increased escape latency caused by A*β*_1−40_ injection at all three test days (day 17 to 19), and the latency reached the level as short as that of normal control. These results indicated that A*β*_1−40_-induced impairments in spatial learning memory were reversed by posttreatment with YCTMT in a dose-dependent manner.

The beneficial effect of YCTMT on A*β*_1−40_-induced learning deficit was further analyzed using probe and working memory tests. The number of annulus crossing, referred to the number of passing over the previous platform site, was measured at day 20. Result showed that number of annulus crossings in A*β*_1−40_-treated group was significantly lower than that of control group, whereas YCTMT posttreatment significantly elevated the number in a dose-dependent manner and showed significance at 1.0 and 2.0 g/kg/day (*P* < 0.05, Figure 3(b)). Compared to the control group, A*β*_1−40_-treated group also showed significantly longer escape latency to found the platform at a constant location and to find the hidden platform at a new location during the reference memory test (day 17–19, Figure 3(a)) and during the working memory test (day 21, Figure 3(c)), respectively, suggesting the A*β*_1−40_-induced impairments in reference and working memory in injected rats. Posttreatment with YCTMT at dose of 1.0 and 2.0 g/kg/day significantly reversed A*β*_1−40_-caused increase of escape latency. These results again demonstrated that YCTMT was able to ameliorate A*β*_1−40_-caused impairment in learning and memory in a variety of forms.

### 3.3. YCTMT Significantly Decreased A*β*_1−40_-Induced A*β* Plaque Area

**Figure 4:**
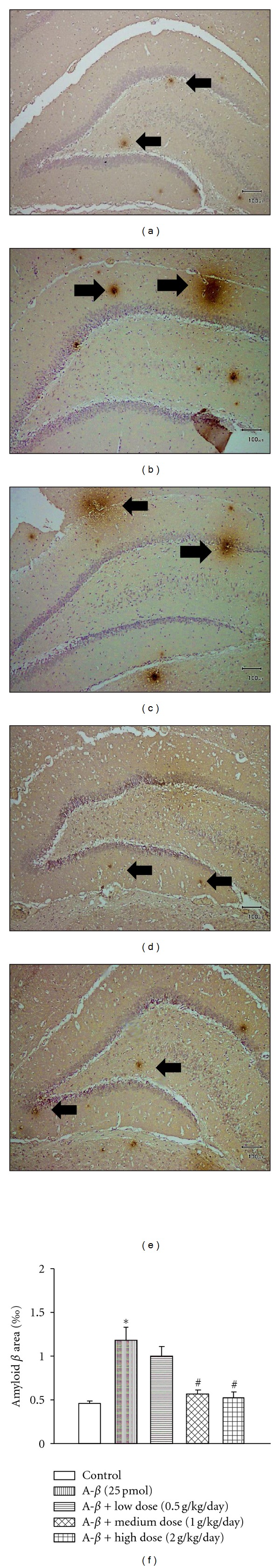
YCTMT posttreatment decreased A*β*_1−40_-induced amyloid accumulation. (a) Control. (b) A*β*_1−40_ infusion (25 pmol). (c)–(e) YCTMT treatment with doses of 0.5, 1.0, and 2.0 g/kg/day. (f) Quantification of amyloid accumulation area. *Represents significant difference between the indicated and normal control group; ^#^between the indicated and A*β*_1−40_ group, *P* < 0.05. The scale bar represents 100 *μ*m.

To investigate the effect of YCTMT posttreatment on amyloid protein burden in the brain, sections were analyzed with immunohistochemical staining. 4G8 antibody against amino acid residues 17–24 of beta-amyloid was used in the study (Figures 4(a)–4(e)). Intracerebroventricular (i.c.v.) administration of A*β*_1−40 _significantly increased A*β* plaque area (arrow in Figure 4(b)) in hippocampus compared with vehicle injection in the control group (Figure 4(a)). Amyloid accumulation was shown in brown and located in the hippocampus. Treatment with 1.0 and 2.0 g/kg/day YCTMT reduced the amyloid-accumulated area, showing as decreased size of 4G8-positive area in hippocampus (Figures 4(d) and 4(e)). Quantitative analysis showed that YCTMT treatment reduced A*β* plaque area in hippocampus in a concentration-dependent manner (Figure 4(f)). This result suggests that the beneficial effect of YCTMT on A*β*_1−40_-induced impairment in cognition might be mediated by reducing A*β* accumulation in the hippocampus.

### 3.4. YCTMT Significantly Reversed A*β*_1−40_-Induced Decrement of Ach Area

**Figure 5:**
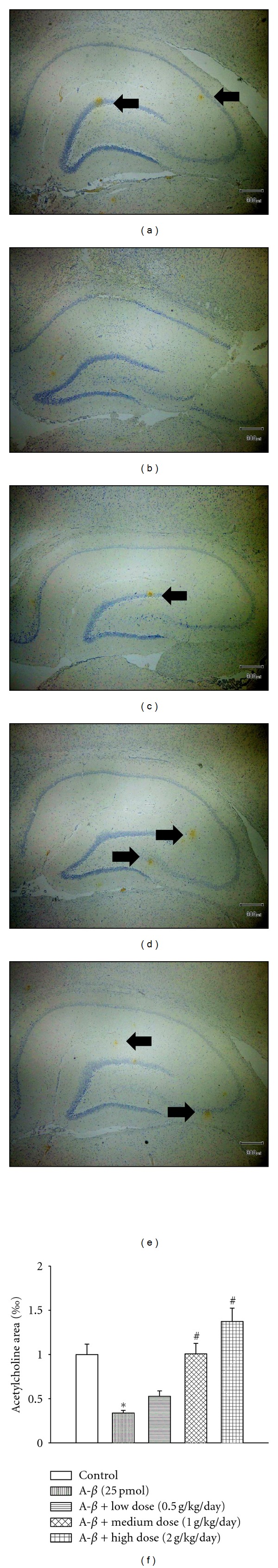
YCTMT posttreatment reversed A*β*_1−40_-induced decreases of Ach level. (a) Control. (b) A*β*_1−40_ infusion (25 pmol). (c)–(e) YCTMT treatment with doses of 0.5, 1.0, and 2.0 g/kg/day, respectively. (f) Quantification of Ach expression. *Represents significant difference between the indicated and normal control group; ^#^between the indicated and A*β*_1−40_ group, *P* < 0.05. The scale bar represents 100 *μ*m.

**Figure 6:**
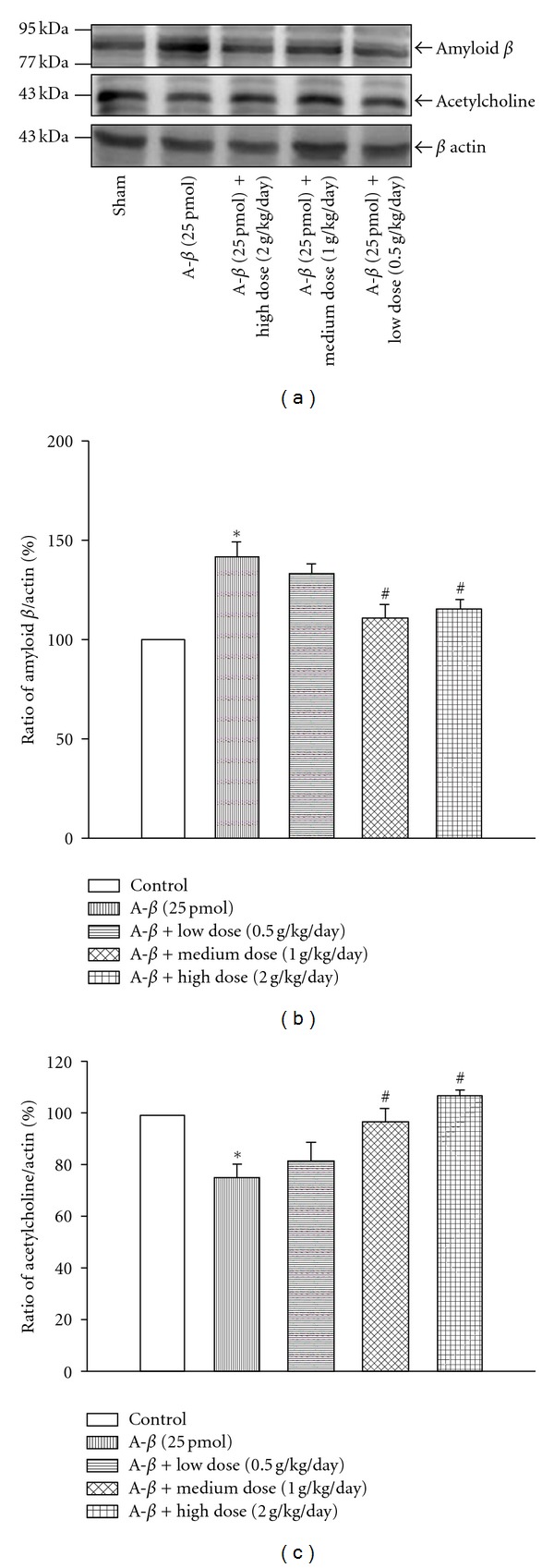
YCTMT posttreatment reversed A*β*_1−40_ and Ach protein level. (a) Comparison of protein level of beta-amyloid and Ach using Western Blot. (b) Quantification of amyloid protein level. (c) Quantification of Ach protein level. *represents significant difference between the indicated and normal control group; ^#^between the indicated and A*β*_1−40_ group, *P* < 0.05.

Ach is an important neurotransmitter and plays critical roles in the formation of learning/memory and etiology of Alzheimer's disease [47]. To explore the possible mechanism of YCTMT-mediated beneficial role in relieving AD-like symptom, we further evaluated Ach expression using immunohistochemical analysis (Figure 5) and Western Blot (Figure 6). Injection of A*β*_1−40_ significantly decreased Ach expression in hippocampus compared with the control group (Figures 5(a) and 5(b)). Quantitative data showed that YCTMT reversed Ach area in a concentration-dependent manner in the whole brain. Treatment with 1.0 and 2.0 g/kg/day YCTMT significantly reversed the expression of Ach in the brain (Figures 5(d) and 5(e)). YCTMT-induced reversal of Ach expression level after A*β*_1−40_ injection was also confirmed with Western Blot. Quantitative result of Western Blot showed that medium- and high-dose YCTMT treatment rescued reduced Ach expression (Figure 6(c)), suggesting the beneficial effect of YCTMT on AD-like symptom. This result suggested that posttreatment with YCTMT could reverse A*β*_1−40_-reduced diminish of Ach expression correlated with functional deficit in the AD brain.

We also found that there was a correlation between amyloid plaque area and acetylcholine expression site (R = 0.719).

## 4. Discussion

In this study, we observed a beneficial effect of YCTMT posttreatment on A*β*_1−40_-induced AD-like symptoms. YCTMT treatment decreased A*β*_1−40_-induced amyloid burden and reversed declined Ach level in hippocampus. It also improved learning and memory function in treated rats in a dose-dependent manner. These results provide evidence of the therapeutic effect of YCTMT on AD and suggest a potential treatment strategy.

Investigators have established that toxic A*β* is the major player in neuronal damage and dementia in both in vitro culture assays and in the intact brain of animal [48]. Notably, i.c.v. injections of A*β*_1−40_ into rats produced learning disability, brain morphological changes, and cholinergic neuronal degeneration [49]. In addition, A*β*_1−40_ induces spatial learning and spatial working memory deficits in animal model [50]. In the present study, we confirmed that continuous infusion of A*β*_1−40_ into the cerebral ventricle induced deficits in memory retention, spatial learning memory, probe memory, and working memory in the rats. Moreover, posttreatment with YCTMT reversed various types of learning and memory loss caused by A*β*_1−40_. These results indicated that YCTMT might have beneficial anti-Alzheimer's disease effects on A*β*_1−40_-induced AD animal model.

We also found that i.c.v. administration of A*β*_1−40_-induced A*β* plague formation was consistent with previous report [15, 18–20, 22]. Both WB and immunostaining results showed that posttreatment with YCTMT for 7 days downregulated A*β* burden in the rat brain. A*β*_1−40_ contributes to the progression of AD and directly impairs cholinergic signaling and Ach release [51]. Activities of choline acetyltransferase and acetylcholinesterase, one for synthesis and the other for hydrolysis of acetylcholine, decrease significantly and correlate with the extent of intellectual impairment in Alzheimer's dementia patients [52]. Coincidently, we also found that posttreatment with YCTMT recovered the expressions of Ach in the rat brains that were challenged with A*β*_1−40_. These results implied that YCTMT might reduce the production of A*β* plaque, possibly through increasing the expression of Ach. However, the mechanisms underlying the influence of Ach expression need further study.

Traditional Chinese Medicine (TCM) has been around for thousands of years and extensively used in the prevention, diagnosis, and treatment of diseases in China. There are several TCMs that have been used to enhance cognitive function and senile dementia [53]. YCTMT is one of the TCMs and has been conventionally used in the improvement of visual acuity and hearing in purpose. Our results provided new evidence of the beneficial role of YCTMT in AD-associated learning and memory deficits. Oral administration of YCTMT in S.D. rats appeared to reverse the A*β*_1−40_-impaired memory, spatial learning memory, probe memory, and working memory deficits. YCTMT might inhibit A*β* plague accumulation and reverse Ach decline. This study suggests a potential use of YCTMT as a potential therapeutic agent for treating AD.

## 5. Conclusion

We have shown that YCTMT inhibits A*β* plague accumulation and rescues reduced acetylcholine expression. This study has provided evidence on the beneficial role of YCTMT in ameliorating amyloid-induced AD-like symptom, indicating that YCTMT may offer an alternative strategy for treating AD.
